# Implication of explicit knowledge and subjective evaluation in procedural perceptual-motor learning

**DOI:** 10.3389/fnbeh.2025.1567905

**Published:** 2025-04-04

**Authors:** Sarah Seiwert, Elodie Martin, Yannick Lagarrigue, David Amarantini, Lilian Fautrelle, Joseph Tisseyre, Jessica Tallet

**Affiliations:** ^1^ToNIC, Université de Toulouse, Inserm, UT3 Paul Sabatier, Toulouse, France; ^2^Department of Functional Physiological Explorations, University Hospital of Toulouse, Toulouse, France; ^3^Institut de Formation en Psychomotricité, Faculté de santé, UT3 Paul Sabatier, Toulouse, France

**Keywords:** sequence learning, Serial Reaction Time Task, SRTT, visuomotor adaptation, double step pointing paradigm, Target Jumping Task, subjective reports

## Abstract

**Introduction:**

Procedural Perceptual-Motor Learning (PPML) enables the acquisition of new motor procedures and is fundamental for a wide range of human behaviors. While traditional research has focused on task-related characteristics, there is growing interest in individual factors to account for inter-individual differences in PPML. This study aims to investigate the roles of two individual factors related to learners’ strategies and mindsets: (a) explicit knowledge of the task’s characteristics and regularities and (b) subjective evaluation of the task and performance. We hypothesized that (a) participants reporting explicit knowledge of the task would exhibit higher PPML scores compared to those who did not, and (b) PPML scores would be related to subjective evaluation.

**Methods:**

Participants were invited to practice two types of PPML tasks: motor sequence learning assessed by a Serial Reaction Time Task (SRTT) (Experiment 1) and visuomotor adaptation assessed by a Target Jumping Task (TJT) (Experiment 2). After each task, they were asked to answer post-learning questions about (a) their explicit knowledge of the task’s rules and (b) their subjective evaluations, including perceived levels of stress, tiredness, motivation, attention, and perceived progress.

**Results:**

The findings of Experiment 1 revealed that participants reporting explicit knowledge of the SRTT exhibited higher learning scores, which were related to perceived stress and progress. In Experiment 2, participants reporting explicit knowledge of the TJT exhibited lower learning scores, which were related to perceived stress, tiredness, concentration, and progress.

**Discussion:**

This study offers a novel and comprehensive perspective on inter-individual differences in PPML by considering the roles of explicit knowledge and subjective evaluations in two types of PPML tasks. Although further replication and generalization are necessary, the findings provide valuable insights into how learner-task interactions may explain inter-individual differences and highlight the importance of considering participants’ subjective reports research for future studies on PPML.

## Introduction

Procedural perceptual-motor learning (PPML) enables to acquire new motor procedures and is thus fundamental for a wide range of human behaviors (Krakauer et al., 2019; Rosenbaum et al., 2001). This process, which requires an extensive practice, results in robust and relatively persistent learned motor procedures (e.g., Schmidt, 1975). A major goal in PPML research has been to identify optimal conditions for PPML. While individual differences such as the learner’s strategies and mindset have been recognized as essential to the learning process and its outcomes, it remains to further explore their relative implication in understanding individual differences in PPML (Willingham, 1998; Wulf and Lewthwaite, 2016). Firstly, strategies can refer to the ability of the learner to develop an explicit knowledge of the task’s rules (e.g., Willingham, 1998). Secondly, factors related to mindset refer to the subjective evaluation of the task and of the performance (e.g., Wulf and Lewthwaite, 2016). On this basis, the present study aims to test the implication of (a) explicit knowledge and (b) perceived levels of motivation, attention, perceived progress, stress and tiredness on PPML, to provide an integrated understanding of the learner’s strategies and mindset relative to PPML.

In the absence of ‘how to learn’ instructions, PPML can occur implicitly, that is, with no or minimal increase in verbal knowledge of movement performance and without awareness of learning (Kleynen et al., 2014). However, participants can develop an explicit knowledge of the task’s rules that can be assessed by verbal reports about the task’s characteristics and regularities (Destrebecqz and Peigneux, 2005; Maresch et al., 2021; Perruchet and Amorim, 1992). This procedure allows researchers to determine the extent to which explicit and declarative rules of the task have been extracted during the practice of the motor skill. The role of declarative knowledge has been widely investigated in the scientific literature (Destrebecqz and Peigneux, 2005). However, its precise impact on motor performance and skill consolidation remains a subject of debate. A deeper understanding of the role of explicit knowledge in PPML can provide fundamental insights into the mechanisms underlying motor control and learning, shedding light on how different memory systems interact during skill acquisition (Schmidt and Lee, 2019). Moreover, explicit knowledge may facilitate or interfere with motor learning depending on the task complexity, the stage of learning, and the individual differences in cognitive strategies (Fitts and Posner, 1967). Finally, a better understanding of implicit and explicit processes in PPML could be used to optimize motor learning in training or in rehabilitation (Boyd and Winstein, 2001; Steenbergen et al., 2010). Noteworthy is that not all participants develop explicit knowledge, suggesting inter-individual differences in learning strategies (e.g., Cornelis et al., 2016; Hegele and Heuer, 2010; Perruchet and Amorim, 1992; Werner and Bock, 2007; Wijeyaratnam et al., 2022; Willingham et al., 1989).

For participants who reported explicit knowledge, some results suggest that explicit knowledge emerges after only twenty exposures to the sequence, considered an early phase of learning (Perruchet and Amorim, 1992), while other results suggest it requires more trials to gradually develop with practice (Weinberger and Green, 2022). Moreover, the link between explicit knowledge and the learning scores is still unclear. Two main types of PPML have been studied: motor sequence learning, referring to the acquisition of a new sequence of movement (Doyon et al., 2003) and visuomotor adaptation, which refers to the process of learning to adapt to environmental perturbations (Doyon et al., 2003). Regarding motor sequence learning, most of studies reported a positive effect or associations with explicit knowledge (e.g., Cornelis et al., 2016; Perruchet and Amorim, 1992; Stefaniak et al., 2008; Willingham et al., 1989), even if some reported no effect (e.g. Sanchez and Reber, 2013) or even a negative effect (e.g. Jiménez et al., 2006). In contrast, research on visuomotor adaptation shows more heterogeneous results, with findings divided between positive (Hegele and Heuer, 2010; Werner and Bock, 2007)and negative effects or associations (Mazzoni and Krakauer, 2006; Wijeyaratnam et al., 2022). However, the limited number of studies in visuomotor adaptation and variations in experimental protocols in both types of PPML prevent a clear consensus from being established. Given these uncertainties, investigating both types of PPML tasks is essential to gain a more integrated understanding of the implication of explicit knowledge in PPML. All in all, these results suggest that participants can develop an explicit knowledge of the task’s rules during PPML but the effect of explicit knowledge on the learning scores is not clear, and it is possible that the type of task-to-be-learnt matters.

Learners’ mindset could also play a role in inter-individual differences regarding PPML, specifically the subjective evaluation of the task and of the performance. Unlike the literature on explicit knowledge, there is limited research on this subjective assessment, which does not allow to clearly dissociate the two types of PPML.

According to the OPTIMAL (Optimizing Performance Through Intrinsic Motivation and Attention for Learning) theory of motor learning, two main subjective components posit an impact on PPML: motivation and attention (Wulf and Lewthwaite, 2016). As regard to motivation, it has a positive impact on PPML (Galea et al., 2015; Huang et al., 2018; Wächter et al., 2009). As regard to attention, mixed results have been reported (McKay et al., 2024). The OPTIMAL theory also includes the concept of self-efficacy, that corresponds to the learners’ subjective evaluation of their performance (Wulf and Lewthwaite, 2016). Studies reported that asking participants questions about the fluidity of their movements after each trial enhanced PPML (Boutin et al., 2014; Ioannucci et al., 2021). The authors attributed this effect to the benefits of focusing on the movement’s outcome and the associated reduction in cognitive effort (Ioannucci et al., 2021). Two other variables may be associated with PPML: stress and tiredness. Although generally negative in affective valence, existing studies on stress indicate no impact on PPML (Ballan and Gabay, 2020; Tóth-Fáber et al., 2021), or even a positive effect (Hordacre et al., 2016). In contrast, studies on tiredness yield inconsistent findings, showing either positive (Borragán et al., 2016) or a negative (Anguera et al., 2012) effect on PPML. All in all, inter-individual differences in PPML could be linked to the perceived levels of motivation, attention, perceived progress, stress and tiredness.

Based on all these results, the present study addresses two primary research questions: (a) Does explicit knowledge of the task affect PPML? and (b) Is subjective evaluation of the task and of the performance, including levels of stress, tiredness, motivation attention and perceived progress, related to PPML? These questions are addressed for the two types of PPML (Doyon et al., 2003): motor sequence learning in Experiment 1 and visuomotor adaptation in Experiment 2.

We hypothesize that explicit knowledge of the task reported by participants has positive effects on motor sequence learning, i.e., participants who report explicit knowledge of the task will exhibit the higher learning scores (Experiment 1). Considering the more contrasted results in the literature on visuomotor adaptation, either positive or negative effect can be hypothesized (Experiment 2). We also hypothesize that the level of stress, tiredness, motivation, attention and perceived progress will be related to PPML scores in both motor sequence learning and visuomotor adaptation tasks.

## Experiments

### Participants

The present study is part of a larger research project aiming to test the link between PPML with laboratory tasks and ecological tasks (Martin et al., 2025), approved by the Research Ethics Committee of the Toulouse University (CER 2020–320). It comprised two experiments, in which forty-two adults (31 females, mean age: 25.63 +/− 4.99 years, mean laterality quotient: 72.16 +/− 39.4%) were included (same participants as in Martin et al., 2025). Criteria for non-inclusion were uncorrected sensory impairment, a self-reported diagnosis of psychiatric or neurological disorder, or a physical injury affecting motor skills. All participants provided written informed consent to participate after being informed on the experimental procedures.

## Experiment 1

Experiment 1 aimed to test (a) the effect of explicit knowledge of the task on motor sequence learning assessed by an adaptation of the Serial Reaction Time Task (SRTT, Nissen and Bullemer, 1987), considered as a reference for motor learning assessment (Krakauer et al., 2019) and (b) the link between the subjective evaluation of the task, including levels of stress, tiredness, motivation, attention and perceived progress and motor sequence learning.

### Material

The material used was a Dell computer with a 17-inch computer screen and a mouse. The computer had OpenSesame® software (version 3.3.8; Mathôt et al., 2012)^1^ with a landscape display. The screen had a standard resolution of 1920 × 1,080 pixels. The computer’s sound was activated at approximately 50 dB.

### Procedure

The experiment was conducted in a quiet room without any potential distractor. This experiment lasted approximately 20 min. After providing informed consent, the participants completed the Oldfield laterality inventory (Oldfield, 1971) to determine their preferred hand that would be used for the two experiments.

#### Task and practice organization

**Figure 1:**
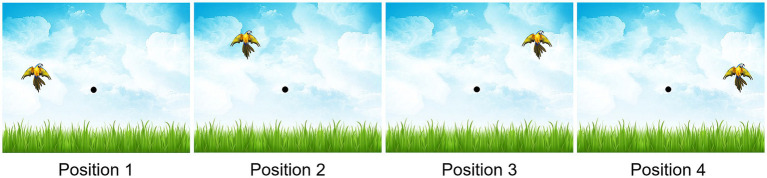
SRTT-like task interface and the four possible stimulus positions.

Participants performed a task inspired by Serial Reaction Time Task (SRTT), a widely used paradigm in the scientific literature to assess motor sequence learning. Unlike traditional SRTT paradigms, which typically involve touch-based responses, this version required participants to use a computer mouse as the response tool. This modification allows to assess the precision of the response using the length of the trajectory in addition to the speed of the response. Moreover, Chambaron et al. (2008) demonstrated the robustness of SRTT learning in adults despite variations in motor response modalities, showing that the use of a mouse did not affect learning compared with the use of a keyboard. The task consisted of one familiarization block and five learning blocks, each comprising 48 stimuli. At the end of each block, participants could take a break and written feedback was provided on the screen concerning the median movement time and the percentage accuracy of the block (percentage of trials without click errors). Participants had to click with the mouse in the center of a bird measuring 2.5 cm high and 3 cm wide. The bird was selected for its potential applicability in studies involving children or individuals with motor impairments. The stimulus could appear at four possible positions arranged in a circular arc (Figure 1). Unknown to the participants, the first three learning blocks as well as the fifth were sequential blocks composed of a sequence of 8 positions of the stimulus repeated 6 times: 2-3-1-4-2-1-3-4. This sequence was designed to ensure equal representation of all positions and to avoid patterns such as repetitions (e.g., 2-2), consecutive ascending or descending chains (e.g., 1-2-3 or 3-2-1), and returns (e.g., 1-3-1). The fourth block was a random block in which stimuli were presented in no specific order but following the previous rules of construction. The following instruction was regularly repeated between learning blocks: “You have to click on the body of the bird as soon as it appears, as quickly and accurately as possible.” Following participant’s correct response or after a maximum duration of 5,000 milliseconds (ms) without a response, the next stimulus appeared after an interval of 200 ms. The familiarization block was composed of 48 stimuli presented in a random order. The five-block organization of the task allowed the identification of four learning phases:

1. A general learning phase composed of the first three sequential blocks, reflecting initial learning of the sequence.
2. A specific learning phase composed of the third sequential block and the random block (Block 4), assessing the effect of the variation in practice.
3. A retention phase composed of the random block (Block 4) and the final sequential block (Block 5), examining the ability to retain the learned sequence.
4. A “resistance to interference” phase composed of the third sequential block and the final sequential block, assessing the evolution of performance before and after the interference of the random block.

These learning phases were selected based on the scientific literature regarding SRTT. The first three learning phases, that is general and specific learning, and retention, were initially described by Knopman and Nissen (1991). General learning reflects overall improvement, specific learning focuses on learning of a specific sequence, and retention tests sequence retrieval. Finally, the resistance of interference phase focuses on changes before and after the variation in practice conditions (Brashers-Krug et al., 1996). The integration of these learning phases enables a comprehensive understanding of the dynamic nature of the learning process.

At the end of the SRTT, participants answered the following questions.

For the explicit knowledge assessment, verbal reports were used to assess the task’s characteristics knowledge with the question: (1) “In how many places did the bird appear?” and the task’s regularities knowledge with the question: (2) “In your opinion, did it appear in a specific order?”

**Figure 2:**
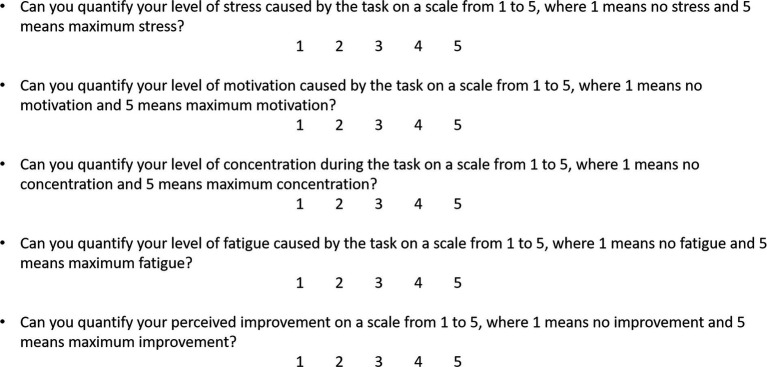
Subjective evaluation of the task and of the performance.

For the subjective evaluation, participants rated their level of stress, tiredness, motivation, concentration and perceived progress on a Likert type scale from 1 to 5 in which 1 meant “not at all” and 5 meant “a lot,” illustrated in Figure 2.

### Data analysis

The laterality quotient (Oldfield, 1971) was calculated using the following formula: (R−L)/(R + L) x 100 where R represents the total points assigned for the right hand and L the total points assigned for the left hand. Points were allocated as follows: 2 points for a hand when used exclusively, 1 point for a hand when used preferentially but with occasional use of the other hand, and 1 point for each hand when both were used without a clear preference.

The movement execution time for each stimulus, the number of click errors before reaching the stimulus and the cursor position every 10 ms for each stimulus were recorded using OpenSesame software (see footnote 1) (version 3.3.8; Mathôt et al., 2012). Data of each block were analyzed using custom Matlab (version R2023b; The MathWorks 2023, Natick, Massachusetts)^2^ scripts to calculate for each block a median time (in ms) from the movement times of all the stimuli of the block. Trials were removed from the analysis if participants made three incorrect clicks near the bird or did not attempt the bird within 5000 ms. These scripts also calculated for each stimulus the length of the mouse trajectory between the coordinates of the starting point to those of the stimulus to be reached from the horizontal (x) and vertical (y) positions. As the x and y coordinates of the mouse were recorded every 10 ms, the length of the segment separating each coordinate was calculated. Some aberrant coordinates caused by mouse lifts were identified and corrected by interpolating segments longer than 250 pixels with adjacent segments. The total trajectory length for each stimulus was then computed by summing these segments. Finally, the script calculated for each block a median length (in pixels) from all the stimuli of the block.

We then computed learning scores for each learning phase. Each learning score corresponds to the difference of the time or trajectory between two blocks, representing the change in performance between two blocks. Four scores were calculated on movement times and trajectories, corresponding to the four phases delimited previously.

1. A general learning score (B1 – B3): a large and positive score means that there is a large difference of time and/or trajectory between blocks 1 and 3 and that participants have reduced their movement time and/or trajectory compared to the beginning of the learning process.
2. A specific learning score (B4 – B3): a large and positive score means that there is a large difference of time and/or trajectory between blocks 3 and 4 and that participants have increased their movement time and/or trajectory when the random block 4 is introduced. This score reflects more specifically the learning of the sequence.
3. A retention score (B4 – B5): a large and positive score means that there is a large difference of time and/or trajectory between blocks 4 and 5 and that participants have reduced their movement time and/or trajectory in block 5. It means that sequence learned during the first three blocks is quickly retrieved in memory when it is reintroduced in block 5.
4. A resistance to interference score (B3 – B5): a score closes to zero means that there is a small difference of time and/or trajectory between blocks 3 and 5. It means that the fourth block of variation had little to no interference with the learning.

**Figure 3:**
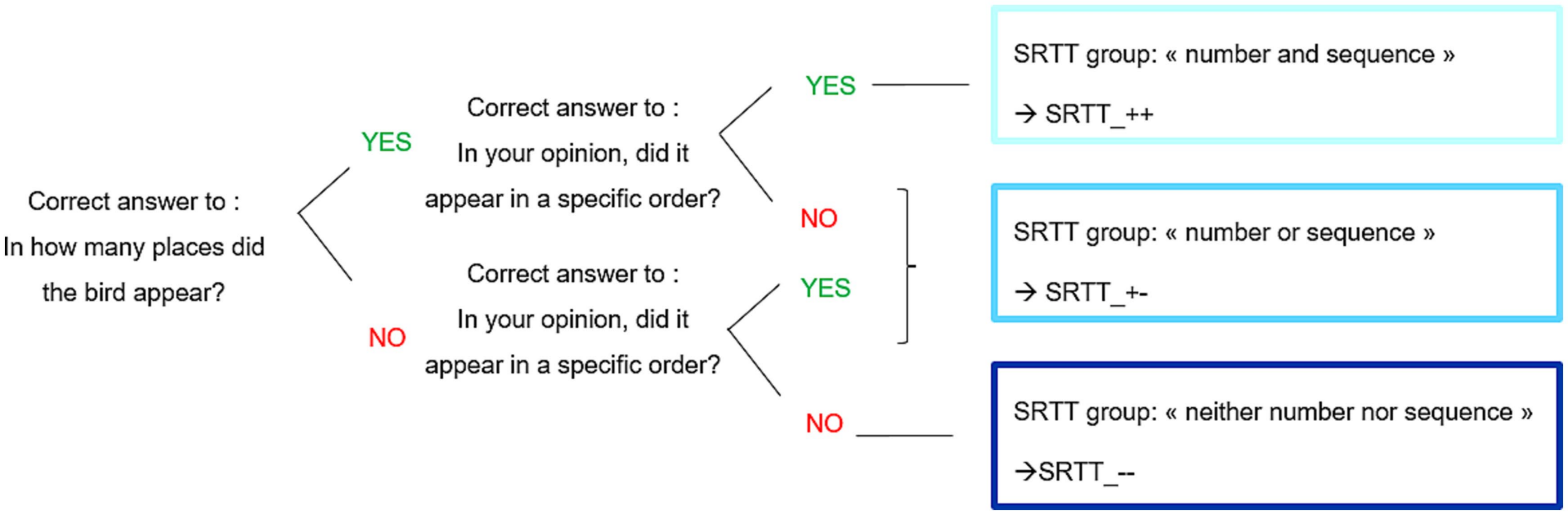
Constitution of subgroups in Experiment 1.

For the analysis of the questions about explicit knowledge, three subgroups were composed according to participant responses to the two questions. The Figure 3 detailed the constitution of the subgroups. The first question was: “In how many places did the bird appear?.” The correct answer was “4,” all other answers were considered as wrong. The second question was: “In your opinion, did it appear in a specific order?.” The correct answer was “yes,” all other answers were considered as wrong. SRTT_++ group was composed of participants who answered correctly to the two questions, SRTT_ + − group was composed of participants who answered correctly to only one of the two question and SRTT_−− group was composed of participants who answered wrongly to the two questions. This approach of subgrouping participants based on post-task responses has been previously employed in PPML research (Hegele and Heuer, 2010; Werner and Bock, 2007).

### Statistics

Statistics were performed with R-Studio (Version 2023.09.1; RStudio Team 2023)^3^ software. If the conditions of normality and homoscedasticity were verified with the Shapiro–wilk and Levene tests, respectively, parametric tests were performed. Otherwise, non-parametric tests were used. The significance level was *p* < 0.05 and effect size will be reported. SRTT data (Experiment 1) were missing for one participant.

To compare group characteristics and performance at the beginning of the Experiments, Chi2 tests were performed between the males/females’ distribution of subgroups and ANOVAs or Kruskal-Wallis tests with a group factor (SRTT_−−, SRTT_ + − and SRTT_++) were computed on ages, laterality quotients and median times and trajectory of the familiarization block (B0). To test the effect of explicit knowledge on PPML, ANOVAs or Kruskal-Wallis with a group factor (SRTT_−−, SRTT_ + − and SRTT_++) were computed on each of the four learning scores, for times and trajectories variables. In case of a significant group effect, Tukey or Dunn’s *post hoc* tests were performed to specify which groups differed.

To test the link between subjective evaluation of the task and of the performance and PPML, Spearman correlations were carried out for all participants between each of the four learning scores, for times and trajectories variables, and levels of stress, motivation, tiredness, concentration and perceived progress. Spearman correlations between levels of stress, motivation, tiredness, concentration and perceived progress were also computed to refine the analyses. If a learning score was significantly correlated with two subjective variables and these two variables were significantly correlated, a linear regression was performed whether there was an interaction between these variables explaining the variation in the learning score.

### Results

#### Prerequisites: groups compositions

The groups were not different in terms of males/females distribution, age, laterality quotient (Oldfield, 1971) and median time and trajectory at the familiarization block (Table 1).

**Table 1:** Males/females distribution, mean age, mean laterality quotient, median time and trajectory at the familiarization block (B0) for Experiment 1.

	Groups			Analysis			
	SRTT_++ (*N* = 20)	SRTT_ + − (*N* = 10)	SRTT_−− (*N* = 11)	*χ*2 (Chi2)	*χ*2 (Krukal-Wallis)	*F* (Anova)	*p*
Male/Female	4/16	3/7	4/7	1.04	–	–	0.60
Age (years)	26.24 ± 5.85	26.77 ± 4.90	23.70 ± 2.91	–	3.16	–	0.21
Laterality quotient (%)	74.80 ± 45.57	77.07 ± 28.80	68.30 ± 50.13	–	0.04	–	0.98
Median time B0 (ms)	1029.88 ± 128.96	1054.45 ± 108.23	1048.14 ± 121.05	–	–	0.16	0.85
Median trajectory B0 (pixels)	581 ± 70.31	559.06 ± 91.30	577.68 ± 41.42	–	–	0.34	0.71

#### Effects of explicit knowledge of the task on PPML scores

**Figure 4:**
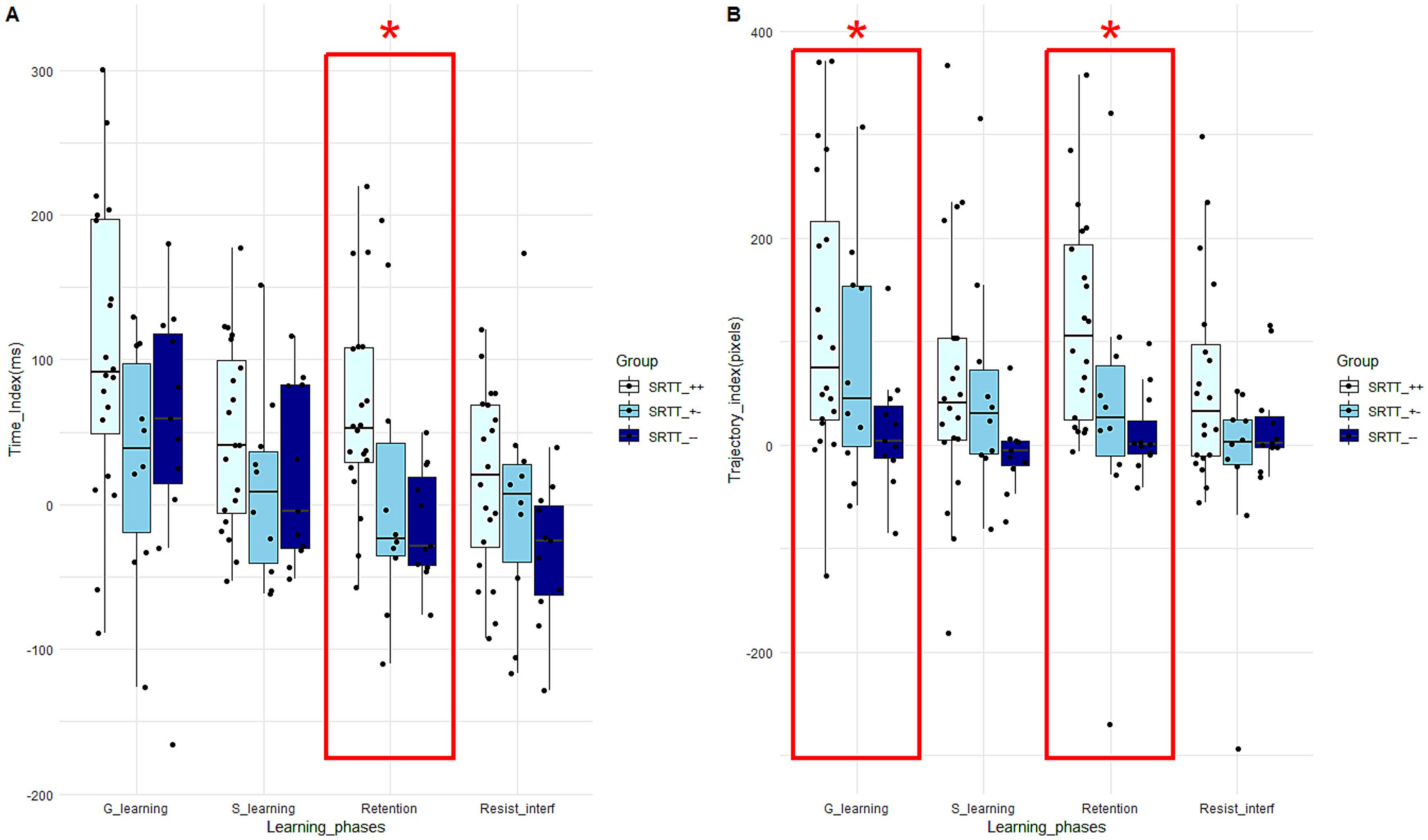
Boxplots of time **(A)** and trajectory **(B)** scores for each learning phase and for the three groups performing the SRTT. The central line represents the median, while the box includes the interquartile range (IQR: Q1 to Q3). Error bars cover to values within 1.5 × IQR. An asterisk “*” means that there is a significant main Group effect (*p* < 0.05) for the learning phase framed in red.

For the median time learning scores, ANOVAs only revealed a significant Group effect for the retention score (*F* (40) = 4.42; *p* = 0.02; *η*^2^ = 0.19). Tukey’s *post hoc* test revealed that the learning score of the “SRTT_++” group (63.68 +/− 69.67) is significantly higher than that of the “SRTT_−−” group (− 13.73 +/− 39.375) (*t* (30) = 2.84; *p* = 0.02; *d* = −1.08) (Figure 4A), suggesting a larger decrease in movement time between the random block and the repeated sequential block. For the median trajectory learning scores, Kruskal-Wallis tests revealed a significant Group effect for the general learning score (*χ*^2^ (40) = 6.24; *p* = 0.04; *ɛ*^2^ = 0.16). Dunn’s post hoc test revealed that the learning score of the “SRTT_++” group (120.79 +/− 138.61) is significantly higher than that of the “SRTT_−−” group (14.25 +/− 59.76) (*z* (30) = −2.50; *p* = 0.04; *d* = −0.91), suggesting a larger decrease in movement time between the first and the third sequential blocks. Kruskal-Wallis tests also revealed a significant Group effect for the retention score (*χ*^2^ (40) = 12.00; *p* = 0.002; *ɛ*^2^ = 0.30). Dunn’s post hoc test revealed that the learning score of the “SRTT_++” group (120.33 +/− 102.35) is significantly higher than that of the “SRTT_−−” group (11.765 +/− 40.40) (*z* (30) = −3.34; *p* = 0.002; *d* = −1.26), suggesting a larger decrease in movement time between the random block and the repeated sequence block (Figure 4B).

#### Links between PPML scores and subjective evaluation

Spearman correlations between the SRTT scores and the different subjective evaluation variables revealed five positive significant correlations (Table 2). Firstly, the higher the SRTT median trajectory learning score during retention, the higher the stress level (rho (39) = 0.33; *p* = 0.04; *r*^2^ = 0.11). Secondly, the higher the SRTT median time learning score during general learning, the higher the perceived progress level (rho (39) = 0.43; *p* = 0.005; *r*^2^ = 0.18). Thirdly, the higher the SRTT median time learning score during retention, the higher the perceived progress level (rho (39) = 0.40; *p* = 0.01; *r*^2^ = 0.16). Fourthly, the higher the SRTT median trajectory learning score during general learning, the higher the perceived progress level (rho (39) = 0.49; *p* = 0.001; *r*^2^ = 0.24). Fifthly, the higher the SRTT trajectory learning score during specific learning, the higher the perceived progress (rho (39) = 0.33; *p* = 0.04; *r*^2^ = 0.11).

**Table 2:** The correlations between SRTT learning scores and subjective evaluation variables.

	Time				Trajectories			
	General learning	Specific learning	Retention	Resistance to interference	General learning	Specific learning	Retention	Resistance to interference
Stress	0.03	0.12	0.27	0.15	0.09	0.20	0.33*	0.10
Tiredness	0.04	0.06	0.11	−0.04	0.10	0.10	0.22	0.09
Motivation	0.21	−0.03	0.01	0.06	−0.06	−0.16	−0.04	0.05
Concentration	0.12	0.005	−0.15	−0.14	0.11	−0.06	0.01	0.02
Perceived progress	0.43**	0.30	0.40*	0.15	0.49**	0.33*	0.30	−0.13

Spearman rho; *p < 0.05; **p < 0.01; *N* = 41. Bold values are significant values.

Spearman correlation between the different subjective evaluation variables revealed one positive significant correlation. The higher motivation level, the higher is the concentration level (rho (40) = 0.43; *p* = 0.01; *r*^2^ = 0.15). As motivation and concentration were not found to be associated with PPML, no linear regression analysis was conducted to explore potential interaction effects.

## Experiment 2

Experiment 2 aimed to test (a) the effect of explicit knowledge of the task on visuomotor adaptation and (b) the link between the subjective evaluation of the task, including levels of stress, tiredness, motivation, attention and perceived progress and visuomotor adaptation. Experiment 2 aimed to test the two previous hypotheses on another type of PPML: the visuomotor adaptation. Unlike motor sequence learning, where SRTT is the gold standard standardized laboratory task, visuomotor adaptation lacks a universally accepted task paradigm, and various tasks are employed in the literature (see Krakauer et al., 2019). In this Experiment 2, we have chosen to create a Target Jumping Task (TJT), inspired by the double-step paradigm (see Gaveau et al., 2014; Magescas and Prablanc, 2006) and which aligns with the definition of visuomotor assessment made by Doyon et al. (2003) as moving a cursor with a computer mouse to reach a moving target on a screen. This task also enables to keep the same materials and perceptual-motor characteristics as those used in Experiment 1.

### Procedure

For Experiment 2, the material and the type of stimuli were the same as for Experiment 1. Specificities of this second Experiment 2 consists in practice organization. This second experiment lasted approximately 20 min.

#### Task and practice organization

**Figure 5:**
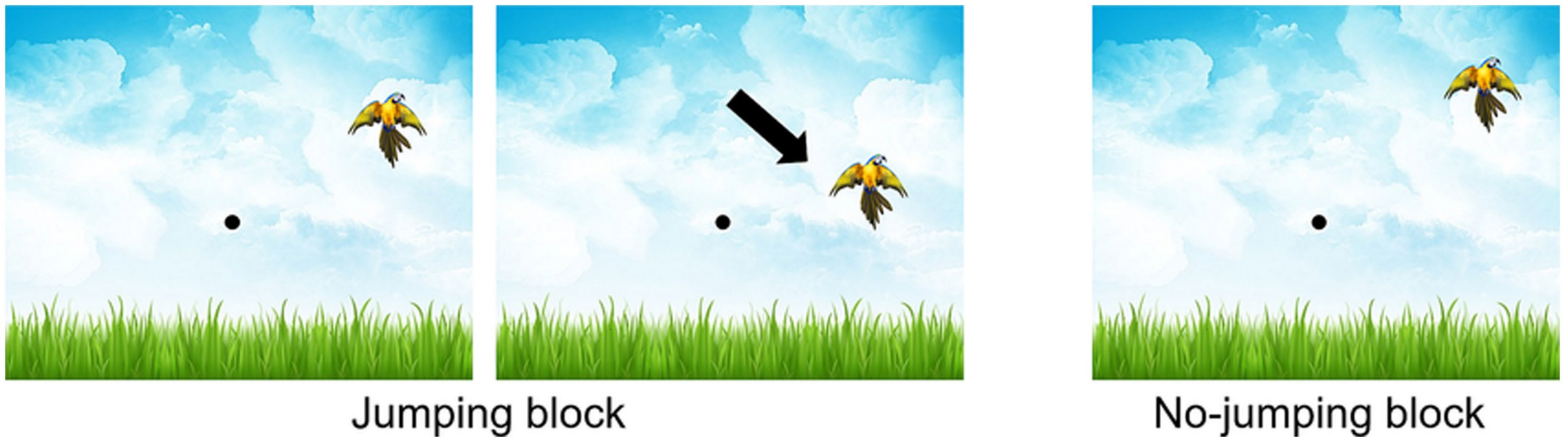
TJT task interface and representation of jumping and no-jumping blocks.

In the TJT, participants first clicked on a central point on the screen to prompt the appearance of the stimulus. Each block (familiarization and the five learning blocks) consisted of 24 stimuli, each trial including a click on the central point and a click on the stimulus (48 clicks per block). After participants clicked on a stimulus or after a maximum response time of 5000 ms, the cursor automatically returned to the central point, requiring participants to click on it to initiate the next trial. The following instruction was regularly repeated between learning blocks: “You have to click on the body of the bird as soon as it appears, as quickly and accurately as possible.” Stimuli were presented at the same four possible positions as in Experiment 1 (see Figure 1) but in a random order in all blocks. In the familiarization block, the stimulus did not move (no-jumping block). In the first three blocks as well as the fifth, the stimulus moved 200 ms after the initiation of the movement (cursor leaving the starting position) by 25° (for stimuli in the right of the screen) or − 25° (for stimuli in the left of the screen) (jumping block). The fourth block was a variation of the practice in which the stimulus did not move (no-jumping block). This variation in practice was selected considering literature assessing visuomotor adaptation by introducing a modified sensorial condition and then remove it to return to a normal sensorial condition (Bastian, 2008; Bo and Lee, 2013). The jumping and no-jumping conditions are illustrated in Figure 5. As in Experiment 1, the five-block organization allowed the identification of four learning phases.

1. An adaptation phase composed of the first three jumping blocks, assessing initial adaptation to the jump.
2. A de-adaptation phase composed of the third jumping block and the no-jumping block (Block 4), reflecting the effect of the variation in practice.
3. A readaptation phase composed of the no-jumping block (Block 4) and the final jumping block (Block 5), assessing the retention of the jump.
4. A resistance to interference phase composed of the third and the final jumping blocks, evaluating the evolution of performance before and after the interference from the no-jumping block.

These learning phases are described in scientific literature regarding visuomotor adaptation and PPML. The first two phases assess adaptation to a modified sensory condition and the de-adaptation to it when return to non-modified sensorial condition (Bastian, 2008; Bo and Lee, 2013). The next two phases allow to see if the adaptation could be retrieval and how the return to non-modified sensory condition affected performance (Brashers-Krug et al., 1996).

At the end of the TJT, participants answered the following questions.

For the explicit knowledge assessment, verbal report was used to assess the task’s characteristics knowledge with the question: “In how many places did the bird appear?”

The subjective evaluation of participants’ level of stress, motivation, tiredness, concentration and perceived progress was similar to that in Experiment 1 (see Figure 2).

### Data analysis

Data analysis was similar to Experiment 1, excepted from the names and the interpretation of the learning scores, as well as analysis of-post task questionnaire, tailored to the specific characteristics of this second experiment. Four scores were calculated, corresponding to the four phases delimited previously.

1. An adaptation score (B1 – B3): a large and positive score means that there is a large difference of time and/or trajectory between blocks 1 and 3 and that participants have reduced their movement time and/or trajectory since the beginning of the learning process.
2. A de-adaptation score (B4 – B3): a score closes to zero means that there is a small difference of time and/or trajectory between blocks 3 and 4 and that adaptation to the jump in block 3 is near to pointing without jump in the block 4. A large and negative score means a more rapid de-adaptation to the jump and/or a weaker adaptation in the first phase.
3. A readaptation score (B4 – B5): a score closes to zero means that there is a small difference of time and/or trajectory between blocks 4 and 5. It means that adaptation to the jump learned during the first three blocks is quickly retrieved in memory when it is reintroduced in block 5. A large and negative score means a slower de-adaptation to the jump.
4. A resistance to interference score (B3 – B5): a score closes to zero means that there is a small difference of time and/or trajectory between blocks 3 and 5. It means that the fourth block of variation had little to no interference with the learning.

**Figure 6:**
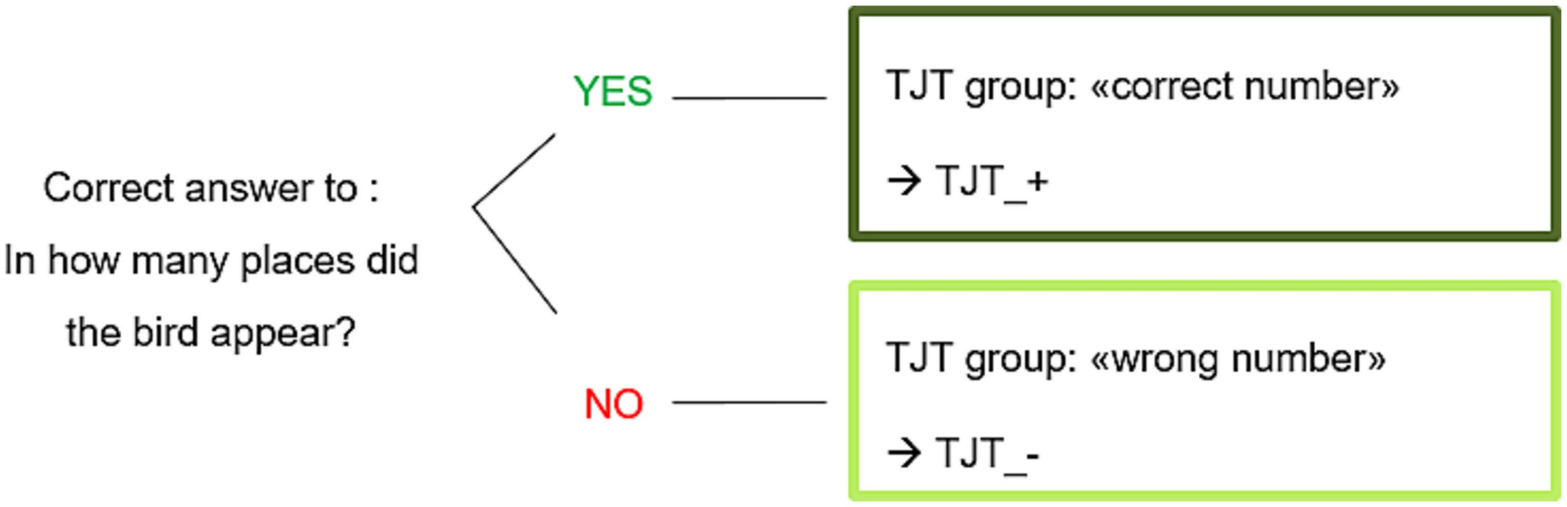
Constitution of subgroups for Experiment 2.

For the question about explicit knowledge, two subgroups were composed according to participant’s response to the question: “In how many places did the bird appear?.” The correct answer was “4,” all other answers were considered as wrong. As illustrated in Figure 6, the TJT_ + group was composed of participants who answered correctly to this question and the TJT_− group was composed of participants who answered wrongly to this question.

### Statistics

As prerequisites, Chi2 tests were performed between the males / females’ distribution of subgroups and Students or Mann–Whitney tests were computed between the two subgroups (TJT_ + and TJT_−) on the same variables as for the Experiment 1.

To test the effect of explicit knowledge on PPML, Students or Mann–Whitney tests were computed between the two subgroups (TJT_− and TJT_+) on each of the four learning scores, for times and trajectories variables.

To test the link between subjective evaluation of the task and of the performance and PPML, Spearman correlations were carried out for all participants between each of the four learning scores, for times and trajectories variables, and levels of stress, motivation, tiredness, concentration and perceived progress. Spearman correlations between levels of stress, motivation, tiredness, concentration and perceived progress were also computed to refine the analyses. If a learning score was significantly correlated with two subjective variables and these two variables were significantly correlated, a linear regression was performed whether there was an interaction between these variables explaining the variation in the learning score.

### Results

#### Prerequisites: groups compositions

The groups were not different in terms of males/females distribution, age, laterality quotient (Oldfield, 1971) and median time and trajectory at the familiarization block (Table 3).

**Table 3:** Males/females distribution, mean age, mean laterality quotient, median time and trajectory at the familiarization block (B0) for Experiment 2.

	Groups		Analysis			
	TJT_+ (*N* = 24)	TJT_− (*N* = 18)	*χ*2 (Chi2)	U (Mann–Whitney)	*t* (T-test)	*p*
Male/Female	6/18	5/13	5.30 × 10^^-31^	–	–	1
Age (years)	26.62 ± 5.88	24.31 ± 3.16	–	165	–	0.20
Laterality quotient (%)	66.07 ± 51.48	76.00 ± 33.09	–	193	–	0.55
Median time B0 (ms)	985.38 ± 102.38	966.25 ± 121.29	–	–	−0.55	0.58
Median trajectory B0 (pixels)	436.04 ± 20.40	446.58 ± 41.36	–	204	–	0.77

##### Effects of explicit knowledge of the task on visuomotor adaptation scores

**Figure 7:**
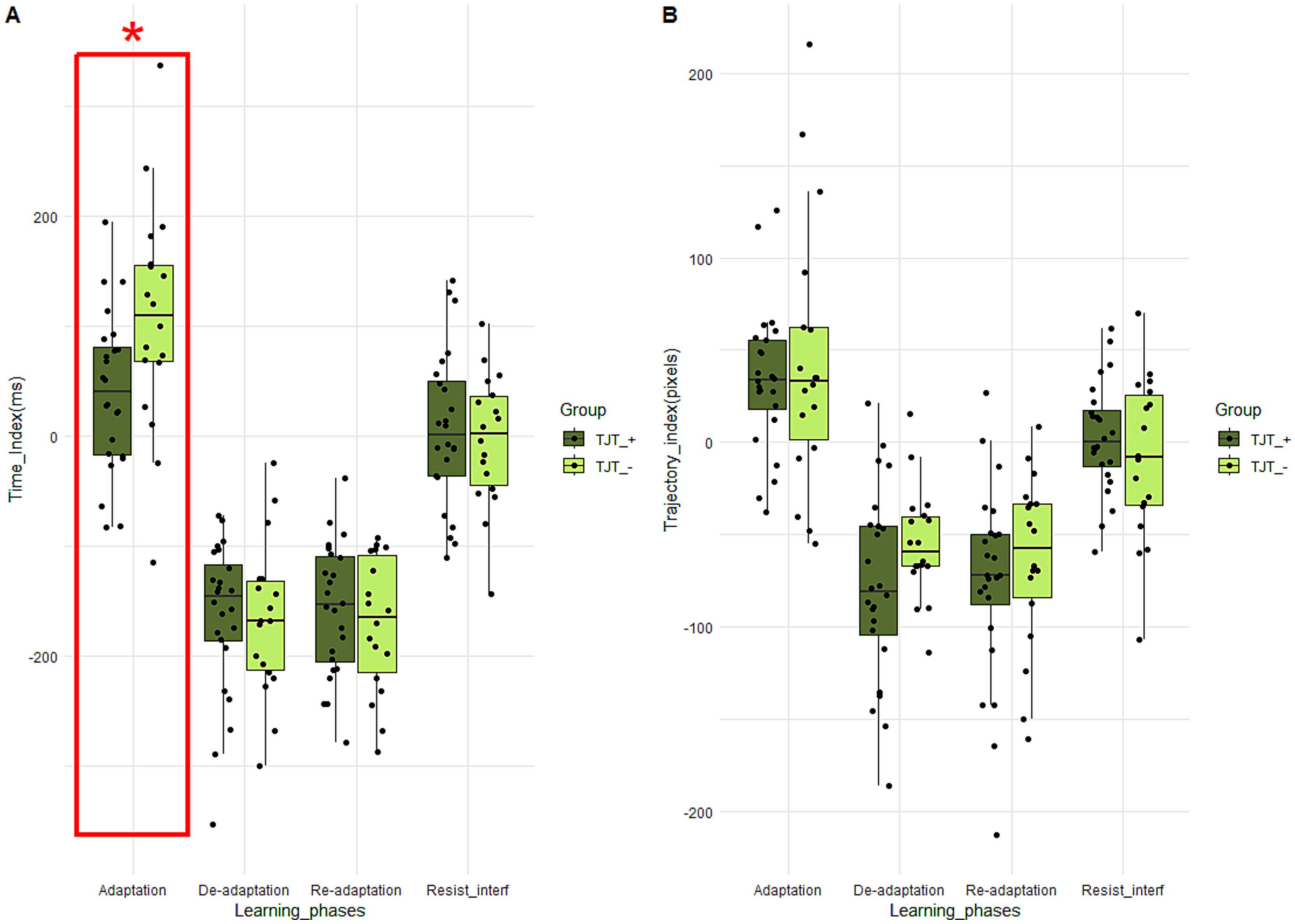
Boxplots of time **(A)** and trajectory **(B)** scores for each learning phase and for the two groups performing the TJT. The central line represents the median, while the box includes the interquartile range (IQR: Q1 to Q3). Error bars cover to values within 1.5 × IQR. An asterisk “*” means that there is a significant main Group effect (*p* < 0.05) for the learning phase framed in red.

For the median time learning scores, Student tests revealed a significant Group effect for the adaptation score (*t* (40) = 2.46; *p* = 0.01; *d* = 0.79). The learning score of the “TJT_− (108.11 +/− 102.36) group is significantly higher than that of the “TJT_+” group (39.88 +/− 71.68), suggesting a larger decrease in movement time between the first and the third jumping blocks (Figure 7A). For the median trajectory learning scores, Mann–Whitney tests revealed no significant difference between “TJT_+” and “TJT_−” groups (Figure 7B).

##### Links between visuomotor adaptation scores and subjective evaluation

Spearman correlations between the TJT scores and the different subjective evaluation variables revealed negative and positive significant correlations (Table 4). Firstly, the lower the TJT median time learning score during adaptation, the higher the stress level (rho (40) = − 0.44; *p* = 0.004; *r*^2^ = 0.19). Secondly, the lower the TJT median trajectory learning score during de-adaptation, the higher the stress level (rho (40) = − 0.38; *p* = 0.01; *r*^2^ = 0.14). Thirdly, the lower the TJT median time learning score during de-adaptation, the higher the tiredness level (rho (40) = − 0.36; *p* = 0.02; *r*^2^ = 0.13). Fourthly, the lower the TJT median trajectory learning score during de-adaptation, the higher the tiredness level (rho (40) = − 0.38; *p* = 0.01; *r*^2^ = 0.14). Fifthly, the higher the TJT time learning score during resistance to interference, the higher tiredness level (rho (40) = 0.34; *p* = 0.03; *r*^2^ = 0.12). Sixthly, the higher the TJT trajectory learning during interference resistance, the higher the concentration level (rho (40) = 0.36; *p* = 0.02; *r*^2^ = 0.13). Finally, the higher the TJT trajectory learning during adaptation, the higher the perceived progress level (rho (40) = 0.31; *p* = 0.04; *r*^2^ = 0.10).

**Table 4:** The correlations between TJT learning scores and subjective evaluation variables.

	Time				Trajectories			
	Adaptation	De-adaptation	Re-adaptation	Resistance to interference	Adaptation	De-adaptation	Re-adaptation	Resistance to interference
Stress	−0.44**	0.12	−0.01	0.16	0.02	−0.38*	0.22	0.17
Tiredness	−0.28	−0.360*	0.03	0.34*	−0.28	−0.38*	0.16	0.21
Motivation	−0.10	−0.05	−0.19	0.13	0.02	−0.04	−0.05	0.10
Concentration	0.08	−0.08	−0.10	−0.07	−0.28	−0.05	−0.30	0.36*
Perceived progress	−0.25	−0.04	−0.18	0.11	0.31*	−0.13	−0.14	0.04

Spearman rho; *p < 0.05; **p < 0.01; *N* = 42. Bold values are significant values.

Spearman correlation between the different subjective evaluation variables revealed two significant positive correlations. Firstly, the higher motivation level, the higher concentration level (rho (41) = 0.33; *p* = 0.03; *r*^2^ = 0.11). Secondly, the higher stress level, the higher tiredness level (rho (41) = 0.35; *p* = 0.02; *r*^2^ = 0.12).

Stress level and tiredness are significantly correlated and both correlated with the TJT trajectory de-adaptation score. Linear regressions were computed but revealed no significant interaction between stress and tiredness levels on the TJT trajectory de-adaptation score.

## Discussion

The present study aimed to explore (a) the impact of the explicit knowledge of the task on PPML and (b) the links between PPML and subjective evaluation of the task and of their performance, including the levels of stress, motivation, tiredness, attention and perceived progress. Experiment 1 assessed motor sequence learning using a Serial Reaction Time Task (SRTT) and Experiment 2 examined visuomotor adaptation through a Target Jumping Task (TJT). At the end of each experiment, participants completed a questionnaire evaluating their (a) explicit knowledge of the task and (b) subjective evaluation of the task and of their performance.

### Effects of explicit knowledge of the task on PPML

In Experiment 1, explicit knowledge of the task positively impacted the learning of the sequence. More precisely, participants with higher explicit knowledge of the task (SRTT_++) at the end of practice were those with better learning outcomes compared to participants with less explicit knowledge (SRTT_−−), both at the beginning (general phase) and at the end (retention phase) of learning. While considering that these groups differences are not related to the age, the laterality or performance in the familiarization block, the observed differences between the groups are consistent with previous research suggesting that explicit processes are involved in implicit learning (Stefaniak et al., 2008; Taylor et al., 2014). Moreover, the observed effects extend findings by Cornelis et al. (2016), who reported a correlation between explicit knowledge of the task and motor sequence learning in various populations. While Cornelis et al. (2016) highlighted this link during specific learning phases, our findings suggest that explicit knowledge of the task positively affects learning both in early and late phases, which is consistent with previous work on the timing of explicit knowledge acquisition during practice in PPML (Perruchet and Amorim, 1992; Weinberger and Green, 2022). However, as the question was asked only at the end of the learning process, we cannot definitively conclude whether explicit knowledge directly influenced learning from the early phases or whether it gradually developed following the learning process. Further studies could assess explicit knowledge at multiple time points during the learning process, as previously done by Weinberger and Green (2022) to clarify whether it is acquired early in learning process or needs further practice to gradually be acquired.

In Experiment 2, explicit knowledge of the task negatively impacted visuomotor adaptation, which is either consistent with some previous studies (Mazzoni and Krakauer, 2006; Wijeyaratnam et al., 2022) or different from others (Hegele and Heuer, 2010; Werner and Bock, 2007). Noteworthy is that our protocol examined the effect of explicit knowledge without influence or controlling for explicit strategies, and provided a naturalistic assessment of how explicit knowledge of the task can interfere with visuomotor adaptation. In that way, it differs from that of Mazzoni and Krakauer (2006) who provided participants with an explicit strategy to compensate the distortion, and that of Wijeyaratnam et al. (2022) who manipulated the way the visuomotor distortion was introduced – both of which could influence explicit knowledge. While considering that the differences in the methods used to assess explicit knowledge can explain the apparent discrepancy of our findings with those from Hegele and Heuer (2010) and Werner and Bock (2007), our results thus extend this existing literature and provide further evidence for the negative effect of explicit knowledge on visuomotor adaptation. In our study, the question was about the number of possible stimuli locations, whereas it was about the distortion of the feedback in previous studies focusing on explicit knowledge of the task. For instance, in a visuomotor rotation task, if the cursor deviates to the left, the participant might consciously decide to “aim to the right” of the displayed target to counterbalance the rotational effect on their motor performance. This deliberate adjustment involves explicitly planning to move to a spatial location distinct from the visual location of the target (Morehead and de Xivry, 2021). Here, the knowledge concerns the locations of the target and not the sensory modification it undergoes. Another interpretation of the negative impact of explicit knowledge on visuomotor adaptation (Experiment 2) could be related to the fact that the TJT was performed after the SRTT (Experiment 1), with a negative effect of learning the sequence in Experiment 1 on adaptation to the jump in Experiment 2. Since the aim of this study was not to compare motor sequence learning and visuomotor adaptation, our results need to be interpreted in light of a possible order effect.

### Links between PPML and subjective evaluation

In Experiment 1, motor sequence learning was related to subjective evaluation, specifically stress and perceived progress. Stress was positively associated to motor sequence learning during late phase of learning. This finding aligns with Hordacre et al. (2016), who reported a positive effect of induced anxiety and stress on learning a grip task. Although the grip task differs from the current study’s task, both involve speed and accuracy. This suggests that the beneficial effect of stress may not be specific to a particular task but could instead reflect a broader mechanism through which stress activates cognitive or physiological processes that enhance learning. Furthermore, although Tóth-Fáber et al. (2021) found no effect of induced stress on PPML, they highlighted a positive influence of stress on detecting probability-based regularities in an task inspired by Serial Reaction Time Task (SRTT), as evidenced by differences in reaction times between random high and random low probability trials. This last result is in line with our results and suggests that stress can improve learning by facilitating the extraction of regularities in the task. To further investigate this hypothesis, we conducted supplementary analyses by testing if SRTT_++ group (which is supposed to have extracted regularities) and SRTT_−− (which is supposed to have less extracted regularities) group differed in terms of subjective evaluation. Results revealed no group differences for stress, suggesting that stress could not explain the explicitness of regularities (see supplementary results). Perceived progress, meanwhile, was linked to motor sequence learning in both the early and late phases of learning. This result may suggest that participants referred to the task as a whole instead of to the last block when they assessed their perceived progress. This interpretation can be linked to the processing of performance feedbacks. Participants received feedback about their median movement time and the percentage accuracy after each block. However, this feedback did not explicitly indicate whether each block was better than the previous one. Even if the interpretation of this feedback could differ among participants, depending on whether priority was given to speed, accuracy or both, our results suggest a consistency between perceived progress and actual results that appears from the earliest phases of learning, and persists during learning. Together, these findings highlight the involvement of cognitive and subjective factors in motor sequence learning, indicating that such influences persist beyond the early stages of learning, contrary to traditional models emphasizing cognitive involvement primarily during initial stages (Anderson, 1982; Fitts, 1954).

In Experiment 2, visuomotor adaptation was related to several subjective variables, including the levels of tiredness, stress, concentration and perceived progress experienced during the task. Moreover, stress and tiredness were positively correlated. Tiredness was negatively linked to visuomotor adaptation at early phase and positively at the late phase of the learning process. Despite differences in protocols, our results in the beginning of the learning agree with those of Anguera et al. (2012) and suggest a negative effect of different type of tiredness on visuomotor adaptation at the early phase of learning. However, while Anguera et al. (2012) specifically focused on cognitive tiredness, the interpretation of tiredness in our study depends on the participants’ perception and could therefore encompass both physical and cognitive tiredness. Supplementary analyses testing if subjective evaluation differed between TJT_− and TJT_ + groups were computed to further our interpretation of our results. Analyses revealed a group difference only for the tiredness (see supplementary results). More tiredness was found for the TJT_ + group compared to the TJT_- group^4^. This increased tiredness may have impacted the adaptation process and could be related to the order in which the two experiments were performed. Since this experiment was performed in second, it could result in higher tiredness, leading to weaker adaptation to the jump and more rapid de-adaptation, especially for the TJT_ + group. Our results also revealed a positive correlation between tiredness and visuomotor adaptation at late phase of learning. This is more surprising when compared with the results of Anguera et al. (2012). In another study, the same authors found effects of individual differences in working memory on visuomotor adaptation only at early phase of learning and not at late phase of learning (Anguera et al., 2010), suggesting that cognitive tiredness could have different effects on visuomotor adaptation in function of the phase of learning. Our results are consistent with this hypothesis, with a negative effect of tiredness at early phases of learning and positive effect at later phases. As regards stress, it is negatively linked to visuomotor adaptation at early phase of the learning process. Moreover, stress is related to tiredness, and both are related to the de-adaptation trajectory learning score. However, multiple regression analyses suggested that stress and tiredness acted independently. Concentration was positively linked to performance during late learning phases, suggesting its importance for sustained adaptation, despite contrasting findings in attentional manipulation studies (e.g., Bédard and Song, 2013). Finally, visuomotor adaptation and the perceived progress are positively linked at the early phases of learning. This result is new in the literature. The early phase of learning is considered as the cognitive stage, during which cognitive factors are more involved (Anderson, 1982; Fitts, 1954; see also Marinelli et al., 2017). At this stage, participants could be more attentive to their progress. Overall, the results for visuomotor adaptation are in line with the involvement of cognitive and subjective factors mainly at the beginning of learning, as reflected by the impact of explicit knowledge and most of the links to subjective evaluation in the early phases. These results are consistent with models that describe a cognitive stage at the beginning of learning (Anderson, 1982; Fitts, 1954).

## General discussion

Globally, our results for the primary aim, the effect of explicit knowledge on PPML, show that explicit knowledge of the task have a positive effect on motor sequence learning (Experiment 1) and a negative effect on visuomotor adaptation (Experiment 2). Our results for the second aim, the link between PPML and subjective evaluation, show a link between sequence PPML and the level of stress and perceived progress (Experiment 1). Visuomotor adaptation is linked to stress, perceived progress, tiredness and concentration (Experiment 2). A central finding emerging from the results of the two experiments is the importance of further investigating the link between PPML-related factors, which may be interrelated. The OPTIMAL theory of motor learning (Wulf and Lewthwaite, 2016) is aligned with this perspective, focusing on motivation and attention effects on motor performance end learning, but also the factors than can influence these processes, such as expectancies and self-efficacy. This kind of theorical model reinforces the idea of studying these factors and their impact on PPML together and not separately. Our results show that attention and motivation are correlated in both motor sequence learning and visuomotor adaptation. However, when analyzing correlations with learning indices, only attention is linked to PPML, more specifically with the visuomotor adaptation in the second experimentation. This choice of scale in five points was made according to literature which often use this number of possibilities (Batterton and Hale, 2017). However, a scale with more possibilities of response could result in a greater variability in responses concerning motivation and concentration, and is therefore a perspective of improvement of subjective evaluation for future studies, providing a clearer idea of the link between these two factors and procedural learning. Another way to increase the precision of this subjective evaluation could be to study its time course by asking these questions at different stage of the learning process. Despite the OPTIMAL theory offered a new perspective of implication with a social-cognitive-affective-motor framework, it considers only a part of factors investigated in our study and these factors are still little studied together in the literature concerning PPML. Expanding this framework to include a broader range of cognitive and subjective factors could enhance our understanding of PPML processes. Our findings also underscore the relevance of metacognition in PPML. Metacognitive models, such as those proposed by Flavell (1979) and Efklides (2008), highlight the interplay between individual awareness and task-related experiences. While traditionally applied to academic learning, these models could provide valuable insights into PPML by exploring how subjective evaluations and explicit knowledge influence learning trajectories.

### Supplementary results

1. Testing groups differences in terms of subjective evaluation

In order to investigate if they were difference of subjective evaluation in function of explicit knowledge, Fisher exact tests were computed. For the SRTT, the tests revealed no differences for any factor of subjective evaluation. For the TJT, the tests revealed only a group difference for tiredness (*p* < 0.001).

### Limits and perspectives

Our results need to be interpreted in light of a possible order effect due to the absence of a counterbalanced design which may have led to interference from motor sequence learning in Experiment 1 on visuomotor learning in Experiment 2. This choice was made because task comparison was not an objective, but this may have interfered with the adaptation and led to greater tiredness in Experiment 2. A first perspective is then to replicate this study using a counterbalanced design to determine whether these findings remain robust regardless of task order. Incorporating brain imaging data could also clarify the implicit and explicit components of motor sequence learning and visuomotor adaptation. As discussed by Mazzoni and Krakauer (2006), brain imaging studies reported that motor sequence learning activated similar left-hemispheric regions regardless of whether the process is implicit or explicit, suggesting a shared neural substrate that could support the acquisition of explicit knowledge from initially implicit learning (Willingham et al., 2002). Moreover, brain imaging studies focusing on hippocampal activation and connection to the striatum during motor sequence learning reported an activation in both implicit and explicit learning and both early and late stages of learning (Albouy et al., 2013; Schendan et al., 2003). Dynamic changes in hippocampal activity may then facilitate the integration and use of explicit knowledge. In contrast, brain imaging studies on visuomotor adaptation reported right-hemispheric activation during implicit adaptation (Krakauer et al., 2004) which could compete with the acquisition of explicit knowledge associated with left-hemispheric activation. Consequently, further brain imaging studies focusing on the implicit and explicit components of motor sequence learning and visuomotor adaptation are necessary to clarify these hypotheses.

A second perspective for further study relates to the assessment of explicit knowledge which relied exclusively on verbal report. While this method is widely used, reproducible, and easy to implement, it may be subject to subjective biases. To refine this assessment, future research could complement verbal reports with additional measures of explicit knowledge, such as recognition tasks, generation tasks or behavioral measures such as speed or error corrections (Destrebecqz and Peigneux, 2005; Maresch et al., 2021).

## Conclusion

Even if some of our findings need to be replicated, they provide an initial overall view of the impact of explicit knowledge and subjective evaluations on motor sequence learning and visuomotor adaptation.

Motor sequence learning is positively affected by explicit knowledge of the task and linked to subjective level of stress and perceived progress. Visuomotor adaptation is negatively affected by explicit knowledge of the task and link to subjective level of stress, tiredness, concentration and perceived progress. These findings are mostly consistent with existing literature on motor sequence learning and provide new insights into visuomotor adaptation that merits further investigation. Further studies based on this approach could provide valuable insights and be extended to educational and rehabilitation contexts where the manipulation of key factors and individualized intervention may optimize the acquisition of new motor skills.

## Data Availability

The raw data supporting the conclusions of this article will be made available by the authors on reasonable request.

## Footnotes

^1^ https://osdoc.cogsci.nl/

^2^ https://www.mathworks.com

^3^ https://www.rstudio.com/

^4^On the Likert scale from 1 to 5, the mean response for TJT_- was 2,44 ± 1,29 while those for TJT_ + was 2,58 ± 0,65.
